# Which Occupation is Highly Associated with Cognitive Impairment? A Gender-Specific Longitudinal Study of Paid and Unpaid Occupations in South Korea

**DOI:** 10.3390/ijerph17217749

**Published:** 2020-10-23

**Authors:** Woojin Chung, Roeul Kim

**Affiliations:** 1 Department of Health Policy and Management, Graduate School of Public Health, Yonsei University, Seoul 03722, Korea; wchung@yuhs.ac; 2 Institute of Health Services Research, Yonsei University, Seoul 03722, Korea; 3 Labor Welfare Research Institute, Korea Workers’ Compensation and Welfare Service, Seoul 07254, Korea

**Keywords:** cognitive impairment, occupation, gender, unpaid work, retired, homemaker, longitudinal, middle-age population, Korea

## Abstract

*Background*: To examine the associations between paid and unpaid occupations and the risk of cognitive impairment with respect to gender in a middle-aged population using the dataset of a nationally representative longitudinal survey. *Methods*: Overall, 24,925 observations of 5865 participants aged 45–64 years were sampled from the seven waves of the Korean Longitudinal Study of Ageing (2006–2018). A dichotomous outcome variable was derived based on the Korean version of the Mini-Mental State Examination scores, and occupations were grouped into 12 categories, including three unpaid ones. Sociodemographics, lifestyle, and medical conditions were included as covariates in the mixed logistic regression models. Adjusted odds ratios and predicted probabilities of cognitive impairment were estimated. *Results:* In the longitudinal models with all-studied covariates, the risk of cognitive impairment was similar between genders but differed across occupation categories for each gender. Moreover, the association between occupation and cognitive impairment varied between genders. Regarding the predicted probability, in men, the retired category exhibited the highest risk of cognitive impairment. However, in women, the highest risk was related to the homemakers category, with the risk being more than five times higher than those in the professionals and related workers category. *Conclusions:* Public health policies to reduce the risk of cognitive impairment in the middle-aged population need to be designed and implemented with respect to both gender and occupation.

## 1. Introduction

Cognitive impairment is defined as a condition in which ‘a person has trouble remembering, learning new things, concentrating, or making decisions that affect their everyday life’ [1]. Cognitive impairment imposes considerable socioeconomic burdens on society, which is likely to increase with the ageing population [2,3]; for example, persons with cognitive impairment have more than three times as many hospital stays as those who are hospitalized for some other conditions [4,5,6]. On average, approximately one-third of people with Alzheimer’s disease or a related dementia are hospitalized at least once annually, and those who are hospitalized at least once have an average of 1.5 to 2 hospitalizations per year [6]. The total number of people with cognitive impairment is estimated to be 75.6 million in 2030 and will nearly triple in 2050 to 135.5 million [7]. Several factors are associated with cognitive function including the following: family history [8], sociodemographics [9,10], lifestyles [11], and health conditions [12,13]. Because most people spend a substantial proportion of their lives at work, it is vital to understand how occupation impacts cognition and what preventative strategies might preserve cognition with ageing. Numerous studies have paid significant attention to occupation, thereby concluding that a person’s occupation (before or after retirement) is significantly associated with that person’s current cognitive function [14,15,16,17,18].

Although previous studies made great contributions to illustrating the association between occupation and cognitive impairment, they had long been subjected to the following four major limitations: (1) a lack of the use of nationally representative samples; (2) a reliance on cross-sectional data; (3) limited categories of occupations; and (4) ignoring unpaid domestic and caring activities. In particular, unpaid work is becoming significantly important in ageing or aged societies because of the increasing number of retired people and informal caregivers [19].

Meanwhile, in a previous study [20], we attempted to overcome these four major limitations in examining the association between occupation and the risk of cognitive impairment. To do so, we used a dataset of a nationally representative longitudinal survey, analysed middle-age population, and considered as many as 10 occupation categories including unpaid work through multivariate, mixed logistic regression model analyses. We subsequently demonstrated that the risk of cognitive impairment differed significantly across occupation categories. The association between occupation and the risk of cognitive impairment was similar between genders, and the highest risk of cognitive impairment was observed in the unpaid domestic and caring activities category, being three times higher than those in the professionals and related workers category. However, the respectful, anonymous reviewers had commented that if the occupations were further categorized into homemakers, retired people, or unemployed people, the association between occupation and cognitive impairment would have differed by gender. Therefore, in the Discussion Section of the previous study, we admitted this point as a limitation of the study and promised to address this issue in detail in the next study.

Evidently, this study is a complement to the previous study, aiming to keep the promise. Using the same dataset of the Korean Longitudinal Study of Aging (KLoSA) survey as in the previous study, adding the recent seventh wave surveyed in 2018 to it, and categorizing unpaid domestic and caring activities into homemakers, retired, or unemployed, we explored the association between occupation and cognitive impairment in detail. We tested the following three hypotheses: (A) the risk of cognitive impairment is similar between genders; (B) the association between occupation and cognitive impairment is similar between genders; and (C) the risk of cognitive impairment is similar across occupation categories. Several considerations led us to test the three hypotheses in the following three models: Model 1, a cross-sectional analysis; Model 2, a longitudinal analysis with no covariate; and Model 3, a longitudinal analysis with all-studied covariates. Accordingly, each of the nine hypotheses was investigated and tested from Model 1-Hypothesis A to Model 3-Hypothesis C. In addition, as in the previous paper, the risk of cognitive impairment was quantified by estimating the predicted probability of cognitive impairment when a person was engaged in a specific occupation and comparing across paid and unpaid occupations.

## 2. Materials and Methods

### 2.1. Data Source and Study Sample

The present study used the same dataset of the KLoSA survey as in the previous paper [20]; additionally, it included the most recent dataset of the seventh wave, thereby using a sample drawn from the first seven waves of the KLoSA survey. The characteristics of the KLoSA survey can be summarised as follows: (1) it is a nationally representative non-institutionalised, civilian population survey conducted biennially from 2006; (2) it uses a stratified, multi-stage, clustered probability sampling design to collect data on Koreans aged 45 years and over living in 15 large administrative areas; (3) it is conducted on behalf of the Korea Employment Information Service under the Korean Ministry of Employment and Labor; and (4) in the baseline survey in 2006 (i.e., the first wave), 10,254 individuals from 6171 households were interviewed using the computer-assisted personal interviewing method [21] on labor-/employment-related, sociodemographic, lifestyle, and health-related characteristics. Detailed information about the survey design and characteristics can be obtained from the KLoSA website (https://survey.keis.or.kr/eng/klosa/klosa01.jsp).

We restricted the present analysis to individuals who were surveyed during the first wave in 2006 to keep the same individuals in the later surveys, collecting 24,925 observations for respondents aged 45–64 years. From these, we excluded the following cases: (1) noncontact, refusal, or death (1146 observations); (2) diagnosis with intellectual disabilities, organic brain diseases, or psychiatric treatments (652 observations); and (3) non-report of the Korean Mini-Mental State Examination (K-MMSE) scores (724 observations). The final study sample is an unbalanced panel sample comprising 5865 participants at baseline and 22,403 observations (men, 9800 observations; women, 12,603 observations) with an average of 3.82 observations per participant (standard deviation = 2.32, range = 1–7). All participants in the KLoSA survey submitted informed consent, conforming to the ethical principles of the Declaration of Helsinki. The Yonsei University Health System Institutional Review Board approved this study (Y-2019–0178).

### 2.2. Measurements

In the KLoSA survey, according to the K-MMSE for cognitive function [22,23], participants were instructed to provide both verbal and written responses to questions about their cognitive function, measuring orientation in time and space, memory, and attention. The total K-MMSE score for each participant was determined, which increased with cognitive function and ranged from 0 to 30. As a dichotomous outcome variable, the K-MMSE score was divided into two [24,25]: a value of 1 (cognitive impairment, K-MMSE score < 24) and 0 (no cognitive impairment, K-MMSE score ≥ 24).

Regarding occupations, different from the previous study where participants who had no job in the labor market (homemakers, unemployed, or retired) were grouped as one category (unpaid domestic and caring activities), in this study, we emphasized the importance of unpaid works worldwide [19,26,27] and grouped participants who had no job in the labor market into the following three categories: (1) homemakers; (2) unemployed; and (3) retired—the KLoSA survey did not provide any information on whether respondents were students or not, probably because it interviewed people aged 45 years and over. Altogether, we considered 12 paid and unpaid occupational categories (nine paid plus three unpaid) as the variables of interest, and the nine paid occupation categories were (4) managers; (5) professionals and related workers; (6) clerks; (7) service workers; (8) sales workers; (9) skilled agricultural, forestry, and fishery workers; (10) craft and related trades workers; (11) plant and machine operators and assemblers; and (12) elementary workers and armed forces.

This study incorporated the following 14 potential covariates: eight sociodemographics (gender, age, marital status, religion, residential area, educational level, household income adjusted for household size, and housing tenure) and six lifestyles and medical conditions (smoking, alcohol intake, routine physical exercise activity, obesity, chronic disease, and depressive symptoms). We defined depressive symptom as a score of four or more on the 10-item short form of the Center for Epidemiologic Studies Depression Scale (CES-D-10). Routine physical exercise activity was assessed according to the information on the participants’ response to a survey question whether the participants engaged in any physical exercise at least once a week for the sake of their own health. Obesity was defined as the body mass index of at least 25 kg/m^2^ based on the revised Asia-Pacific criteria by the World Health Organization of the Western Pacific Region [28]. Chronic disease was defined based on self-reported answers to survey questions, that is, whether a chronic disease (hypertension, diabetes, stroke, angina, myocardial infarction, chronic pulmonary diseases, and any type of cancer) had been diagnosed by a physician.

### 2.3. Statistical Analyses

We considered both because we analysed a longitudinal dataset, participant’s observations were likely to be temporally correlated within the participant and that the dependent variable in the present study was dichotomous. Therefore, as suggested by several studies and textbooks [29,30,31,32], we chose the mixed logistic regression model method as a main analysis tool of our study. To achieve the aim of the present study, we considered the aforementioned three models: Model 1 (a cross-sectional analysis) analysed observations at baseline (Wave 1); Model 2 (a longitudinal analysis with no covariate) analysed all waves of observations adjusted for temporal correlation between observations within a participant but unadjusted for any covariate; and Model 3 (a longitudinal analysis with all-studied covariates) analysed all waves of observations both adjusted for temporal correlation between observations within a participant and adjusted for all-studied covariates. Then, we performed a sevenfold analysis.

First, in deciding the necessity of a mixed model framework in longitudinal analyses for all participants in Models 2 and 3, we examined whether observations were temporally correlated within the same participant and decided to use it: intraclass correlation coefficient (ICC) was 0.62, and its 95% confidence interval was 0.58 to 0.66 in the null model.

Second, because the estimation in a mixed logistic model framework may cause bias in parameter estimates, we scaled the conditional weights at the observation level to sum to the sample size within each participant [33,34].

Third, to test Hypotheses A and B, we sought a good specification for Model 3, a multivariate model analyzing men and women together, with no strong multicollinearity (the value for the variance inflation factor [VIF], <2.03) and no evidence of a lack of goodness-of-fit (*p*-values based on the Hosmer–Lemeshow statistic, 0.782).

Fourth, for Models 1–3, we tested Hypothesis A using the Wald test. For Models 2 and 3, we used only the main-effects terms of gender and occupation variables. Subsequently, in Model 1, the prevalence rate of cognitive impairment in women (67.3%, 95% confidence interval [CI]: 62.9% to 71.4%) was significantly higher than that in men (32.7%, 95% CI: 28.6% to 37.2%). In Model 2, men were less likely than women to have cognitive impairment (odds ratio [OR]: 0.53, 95% CI: 0.38 to 0.74). However, in Model 3, compared with women, men had a similar likelihood to have cognitive impairment (OR: 0.99, 95% CI: 0.80 to 1.24). Therefore, we rejected the hypothesis in Models 1 and 2 (*p* < 0.001 in both), but not in Model 3 (*p* = 0.967). Considering that Model 3 analysed all waves of observations both adjusted for temporal correlation between observations within a participant and adjusted for all-studied covariates, we respected the results of Model 3 and decided not to reject Hypothesis A, thereby suggesting that the adjusted risk of cognitive impairment is similar between genders.

Fifth, in a similar way, we tested Hypothesis B—the association between occupation and cognitive impairment is similar between genders—for Models 1–3 using a Wald test. In Model 1, we tested the hypothesis using a simple logistic model framework with the main-effect terms of the occupation and gender variables together with their interaction-effect term, and in Models 2 and 3, we tested it using a mixed logistic model framework with the main-effect terms of the occupation and gender variables together with their interaction-effect term. Consequently, we rejected the hypothesis in Model 2 (*p* = 0.014) and Model 3 (*p* = 0.023), but not in Model 1 (*p* = 0.142). Emphasizing the results of the two longitudinal models with all longitudinal observations, we rejected Hypothesis B. This suggests that the adjusted association between occupation and cognitive impairment differed between genders; therefore, we decided to stratify all the remaining analyses by gender.

Sixth, for gender-specific analysis to test Hypothesis C, we re-evaluated the necessity of a mixed model framework for Models 2 and 3 and confirmed it from a null model for each gender (ICC: 0.63, 95% CI: 0.56 to 0.69 in men; ICC: 0.60, 95% CI: 0.55 to 0.65 in women). Since Model 3 is multivariable, we sought a model for each gender, which implies no strong multicollinearity (the value of VIF, <2.49 in men; <1.42 in women) and no evidence of a lack of goodness-of-fit (*p*-value based on the Hosmer–Lemeshow statistic, 0.704 in men; 0.768 in women). Subsequently, we tested Hypothesis C for Models 1–3.

Seventh, we estimated the participants’ predicted probabilities of cognitive impairment and their 95% CIs using the delta method [35] if the participants were engaged in a particular occupation and if all other characteristics were held constant at their own values. Then, we tested whether the predicted probability was similar between a particular occupation category and a reference occupation category using the Wald test.

Through all estimation processes, all characteristics were considered time dependent (i.e., could change with time). For mixed logistic analyses, ORs and their 95% CIs were estimated. *p* values < 0.05 (two-tailed) were considered statistically significant. All values were estimated with a complex sampling design. Statistical analyses were performed using the Statistical Analysis System (SAS) version 9.4 software (SAS Institute, Cary, NC, USA) and STATA 15 software (StataCorp, College Station, TX, USA).

## 3. Results

Regarding the characteristics of the sample participants at baseline (Wave 1) by gender, cognitive function (K-MMSE) score was higher in men than in women (median, 29.0 vs. 28.0; *p*-value, <0.001), and age was similar between men and women (median, 54.0 in men vs. 53.0; *p*-value, 0.222) (Table 1).

A significantly higher proportion of men than women was found in the following categories of each characteristic: married; having no religion; attaining an educational level of middle school or high school; attaining an educational level of college or higher; belonging to each occupation category except the homemakers, the retired and the service workers categories; belonging to the higher half group of household income; smoking; alcohol intake; or having no depressive symptoms (Supplementary Table S1 exhibits sample characteristics at waves 2 to 7 by gender).

According to the results from Model 1 (a cross-sectional analysis), cognitive impairment at baseline (Wave 1) was prevalent with almost twice the rate in women (11.5%, 95% CI: 10.5% to 12.7%) than that in men (5.7%, 95% CI: 4.8% to 6.6%) (Rao–Scott chi-squared test, *p* < 0.001) (Table 2).

With respect to occupation categories, the prevalence of cognitive impairment differed across occupation categories for each gender (Rao–Scott chi-squared test, *p* < 0.001). This led us to reject Hypothesis C for each gender in Model 1 that the risk of cognitive impairment is similar across occupation categories.

Regarding the prevalence rate of cognitive impairment, occupation categories exhibiting the two highest values differed between genders: the retired category (14.5%, 95% CI: 10.4% to 19.9%) and the homemakers category (9.2%, 95% CI: 6.2% to 13.4%) in men and the skilled agricultural, forestry, and fishery workers category (22.3%, 95% CI: 15.5% to 31.0%) and the homemakers category (14.1%, 95% CI: 12.5% to 16.0%) in women. On the contrary, occupation categories exhibiting the two lowest values also differed between genders: the clerks category (2.3%, 95% CI: 1.0% to 5.0%) and the managers category (2.9%, 95% CI: 1.4% to 6.1%) in men. However, in women, none of the patients had cognitive impairment in the managers category (52 subjects) and the professionals and related workers category (58 subjects).

According to the results from Model 2 (a longitudinal analysis with no covariate), the risk of cognitive impairment differed across occupation categories for each gender, being more strongly in women (Wald test, *p* < 0.001) than in men (Wald test, *p* < 0.005) (Table 3). This led to the rejection of Hypothesis C for each gender in Model 2. Therefore, this suggests that in Model 2, the risk of cognitive impairment differs across occupation categories for each gender.

The results of Model 3 (a longitudinal analysis with all-studied covariates) showed that the risk of cognitive impairment differed across occupation categories in both men (Wald test, *p* = 0.006) and women (Wald test, *p* < 0.001). Consequently, in Model 3, this led us to reject Hypothesis C for each gender that the risk of cognitive impairment is similar across occupation categories.

However, it is noteworthy that the differences in the OR of cognitive impairment across occupation categories were significantly larger in women than those in men. Compared to the homemakers category, the occupation categories exhibiting the two lowest ORs of cognitive impairment were the professionals and related workers category (OR: 0.34, 95% CI: 0.14 to 0.81) and the sales workers category (OR: 0.52, 95% CI: 0.28 to 0.98) in men; and the professionals and related workers category (OR: 0.10, 95% CI: 0.02 to 0.43) and the plant, machine operators, and assemblers category (OR: 0.30, 95% CI: 0.11 to 0.81) in women.

Regarding the covariates, those with a significant association with cognitive impairment were educational level, alcohol intake, routine physical exercise, and depressive symptoms for both genders, smoking for men only, and household income and housing tenure for women only.

**Figure 1:**
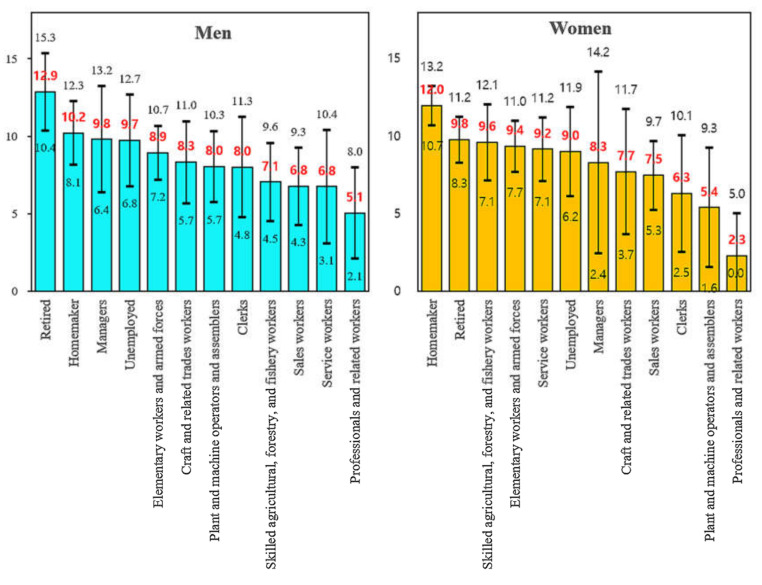
Predicted probability (%) (red color) and its 95% confidence interval of cognitive impairment (black color) by gender and occupation category.

Regarding the predicted probability of cognitive impairment for each occupation category according to the results obtained from Model 3 (a longitudinal analysis with all-studied covariates) (Figure 1), in men, those in the retired category were exposed to the highest risk of cognitive impairment; their adjusted risk of cognitive impairment (12.9%, 95% CI: 10.4% to 15.3%) was more than twice the risk of those in the professionals and related workers category (5.1%, 95% CI: 2.1% to 8.0%), with a significant difference (Wald test, *p* = 0.002). Different from men, women exposed to the highest risk of cognitive impairment were those in the homemakers category; their adjusted risk of cognitive impairment (12.0%, 95% CI: 10.7% to 13.2%) was more than five times the risk of those in the professionals and related workers category (2.3%, 95% CI: 0.0% to 5.0%), with a significant difference (the Wald test, *p* < 0.001).

## 4. Discussion

In this study, we investigated the associations between paid and unpaid occupations and the risk of cognitive impairment in a middle-aged population. The results obtained from a model adjusted for temporal correlation between observations within an individual and for all-studied covariates (Model 3) showed the following five main results: first, the adjusted risk of cognitive impairment was similar between genders; second, the adjusted risk of cognitive impairment varied across paid and unpaid occupations for each gender; third, the adjusted association between occupation and the risk of cognitive impairment differed between genders; fourth, among all studied occupation categories, the retired and homemakers categories in men and women, respectively, had the highest adjusted risk of cognitive impairment, whereas the professionals and related workers category in both genders had the lowest adjusted risk; and fifth, the difference in the adjusted risk between the highest and lowest values across occupation categories was significant, being significantly larger in women than in men.

The finding in this study, that is, the adjusted risk of cognitive impairment was similar between genders in the middle-aged population, seems to be consistent with the results of previous studies in the middle-aged and elderly population in the Netherlands [36] (between 60 and 80 years of age) through a systematic review [37]. However, it should be noted that some previous studies showed different results according to a society’s economic development. In developed countries, compared with older men, older women tend to have a similar or better level of cognitive impairment risk [38,39], whereas in developing countries, older women are likely to have a higher risk than older men [40,41,42,43]. One study addressed why gender differences in cognitive performance in middle-aged and older populations across Europe declined over time [44]. Using data from the Survey of Health, Ageing and Retirement in Europe and after merging 13 countries into three geographical regions, they found that the difference in later cognitive performance between genders was influenced by gender differences with regard to living conditions and educational opportunities to which individuals are exposed during their formative years, along with the gender differences in cognitive tasks, birth cohorts, and regions. In this respect, in Korea, an increase in overall economic prosperity, an increased exposure to cognitive stimulation under a significantly competitive market economy system, a remarkable improvement of population health, and a decrease in the average size of the family in which women would take care seem to partly contribute to narrowing gender differences in the risk of cognitive impairment over time.

In addition, this study showed that the adjusted risk of cognitive impairment differed across paid and unpaid occupations in the middle-aged population. Concerning this, previous studies may be grouped into two categories according to whether participants were investigated before or after retirement. One group comprises studies of middle-aged people before retirement (the association between their current occupation and their cognitive impairment) [14,15]. For example, Van der Elst et al. (2012) used a case-control design, with 50 teachers and 50 non-teacher controls who were matched for level of occupation, educational level, age, and gender taking part in the Maastricht Aging Study (MAAS) in the Netherlands. They found that primary and secondary teachers have better working memory and verbal fluency abilities than participants in other occupations, even when matched for age, gender, occupation, and educational level [14]. The other group comprises studies of the elderly after retirement (the association between occupation before their retirement and their current cognitive impairment) [16,17,45]. For example, Fisher et al. (2014) investigated the course of cognitive functioning before and after retirement and specifically the job characteristics during one’s time of employment using 4,182 individuals in the Health and Retirement study across an 18-year time span (1992–2010) in the United States. Results indicated that working in an occupation characterized by higher levels of mental demand was associated with higher levels of cognitive functioning before retirement and a slower rate of cognitive decline after retirement [16]. A well-accepted, plausible mechanism is the difference in mental demand across different occupations [14,16,46,47,48]. If people engage in occupations with a higher level of mental demand due to greater occupational complexity and patterns of occupational demands before their retirement, it likely leads not only to a higher level of cognitive functioning after retirement through decreased hippocampal volume and increased whole-brain atrophy [14,15] but also to a slower rate of cognitive decline after retirement [16,17,45]. Based on this, the reason why the professionals and related workers category in this study showed the lowest adjusted risk of cognitive impairment for each gender may be partly because their jobs demand high mental activities [18,46,49,50]. Bosma et al. (2003) found that people with mentally demanding jobs had lower risks of developing cognitive impairment compared with people without such jobs [46]. Similarly, using the Leukoaraiosis and Disability (LADIS) study in Europe, Jokinen et al. (2016) showed that people with a cognitive demanding occupation, such as white-collar, professional, or managerial worker, had a slower rate in decline in working memory and immediate memory recall [49].

Why would this study report a gender difference in the adjusted association between occupation and cognitive impairment in the middle-aged population? A tip for the answer may lie in this: a gender difference was found when unpaid works were categorized into homemakers, retired, and unemployed; however, no gender difference was found when unpaid works were not further categorized in the previous study [20]. In the previous study, the risk of cognitive impairment in the unpaid housekeepers category (11.2%) was the highest among occupation categories, being three times higher than those in the professionals and related workers category (3.7%). Therefore, it is evident that detailed categorization of unpaid works leads to gender differences in the adjusted association between occupation and cognitive impairment. Accordingly, we established two hypotheses: one is the hypothesis of ‘voluntary choice’, and the other is the hypothesis of ‘involuntary dispatch’. The hypothesis of voluntary choice is based on the notion of sociology and labor economics [51,52]. In most societies, whether male-dominated or not, men are likely to be primary earners, but women are likely to be non-earners or secondary earners, and after marriage, women tend to become unpaid homemakers or quit jobs more often than do men. Moreover, it is a well-known fact that the welfare system in Asia, which has heavily depended on family members (mainly women), has provided a strong impetus for rapid economic development [53]. Therefore, it may be thought that the distribution of occupations—more precisely paid and unpaid occupations—may not be similar between married men and women. To prove this, using the study sample in this study, we tested a subordinate hypothesis that the distribution of paid and unpaid occupations was similar between married men and women and rejected it (design-adjusted chi-squared test for each wave, p-value <0.0001). Therefore, this suggests that the distribution of paid and unpaid occupations differs between married men and women, thereby supporting the hypothesis of voluntary choice.

In addition, we paid attention to the findings that in male-dominated societies, the freedom of occupation choice is unlikely to be granted to women [54,55]. For example, a study of 36,000 job adverts in China revealed that 56% of China’s National Bureau of Statistics was listed as male-preferred or male-only compared with 25% of job postings at the Civil Aviation Administration. Women-only jobs listings were typically for housekeeping, child care, or administrative assistants [56]. Therefore, the hypothesis of the involuntary dispatch that we established may state that in male-dominated societies, women without cognitive impairment are not as free to choose their occupations as their male counterparts are. Using the study sample in this study, we tested if the distribution of occupations was similar between men and women with no cognitive impairment and rejected it (design-adjusted chi-squared test for each wave, *p*-value < 0.0001). This may suggest that in male-dominated societies, such as Korea, the freedom of occupation choice differs between genders, thereby leading to a gender difference in the adjusted association between occupation and cognitive impairment. Although Korea is the 12th largest developed economy in the world [57,58], it ranked 108th in the Gender Gap Index 2020 among 153 countries [59]; women’s difficulties in male-dominated labor markets in Korea are well documented in other studies [53,60,61,62,63,64]. However, because the two hypotheses (voluntary choice and involuntary dispatch) were first introduced in this study, they need to be examined more rigorously in future studies to account for the gender differences in the associations of occupation with cognitive functioning.

In this study, the retired category in men and the homemakers category in women were associated with the highest risk of cognitive impairment. For adult men, it seems that irrespective of their cognitive ability, they, as primary earners (whether salaried or self-employed), are required to get paid jobs and keep on making money for themselves or their family members, and some of them may exit the labor market owing to various problems, including cognitive impairment. In contrast, for women, as secondary earners, a ‘segregation phenomenon’ may occur based on cognitive ability: women with a higher level of cognitive functioning are likely to be eager to get the most competitive, paid occupations such as the professionals and related workers category. However, women with a lower level of cognitive functioning might remain in the least competitive occupations such as the homemakers category from their early adulthood or enter the labor market but quit their jobs (whether voluntarily or involuntarily) more often than do their male counterparts. Therefore, it seems that this segregation phenomenon and the ‘use it or lose it’ perspective on cognitive ageing [16,46,65,66,67,68] may widen during ageing the difference in the adjusted risk of cognitive impairment between women in the professionals and related workers category and those in the homemakers category. Undoubtedly, this segregation phenomenon would be severe in male-dominated societies. Interestingly, in this study of Korea, in the professionals and related workers category, the women’s adjusted risk of cognitive impairment (2.3%, 95% CI: 0.0% to 5.0%) was less than one-half of that in men (5.1%, 95% CI: 2.1% to 8.0%) in terms of the predicted probability of cognitive impairment. This may imply that in the professionals and related workers category in the male-dominated labor market of Korea, women are likely to work with a higher level of cognitive functioning than are men, and this may cause highly educated women to get stressed [61]. Future studies need to investigate this segregation phenomenon and its resulting harm to individuals and society more precisely.

Although drawing policy suggestions based on the results of this study requires substantial caution, we believe that public health policies toward population ageing need to identify a group or population that is or will be exposed to a high risk of cognitive impairment and design a variety of policies to reduce their risk. Therefore, in this study, we identified the highest risk groups for each gender (the retired in men and the homemakers in women). Previous studies implied that retired people or life-long homemakers may be exposed to a faster cognitive decline compared with those engaged in more mentally demanding work [16,46,65,66,67,68], mainly because the unpaid work—childcare, housework, and home maintenance—of retired people and homemakers are related to less complex environments with minimal cognitive loads [69,70,71] and less protective of cognitive function [48,72]. For instance, Leist et al. (2013) found that periods away from work described as unemployment in adult life are associated with lower cognitive function at old age. They assumed that adults with temporary unemployment reported as sickness or homemaking may not achieve the socioeconomic stability with stable work trajectories, which in turn may influence later-life cognitive function [73]. Therefore, these people need to be encouraged to do work that may help maintain intellectual stimulation; performing volunteer work could be an option for activating cognitive function [74]. In addition, numerous studies have found that social connectedness and loneliness influence older people’s cognitive impairment [43,74,75]. Feelings of being connected to their family and community may be as important as new medicines or therapies that can reduce symptoms associated with cognitive decline for maintaining good cognitive function in older age [75]. For instance, a study of UK reported that transportation policies (free bus travel) may increase the elderly’s cognitive functions through participation in volunteering activities and contact with older people’s adult children and friends [76]. Therefore, social policies for the retired and homemakers are needed to reduce the risks of cognitive impairment such as lifelong learning, volunteer work, and participation in social and leisure activities either at home or in community centers [43,77].

Meanwhile, regarding educational level, we found that higher educational level was associated with lower risk of cognitive impairment for both genders. These findings add to a growing body of literature, indicating that education may reduce cognitive decline [78,79,80]. However, the exact nature of the association between educational level and cognitive function is complex. A possible explanation is that education improves cognitive function [81]. Another possibility is that people with higher cognitive function seek and gain access to longer education [80].

In addition to the strengths of the previous study [20], this study has two strengths. First, to explore in detail the association between occupation and the risk of cognitive impairment, we divided all the people who were engaged in unpaid domestic and caring activities into three categories: homemakers, retired individuals, and unemployed people. Second, we included a recent seventh wave of the KLoSA survey and analysed its first seven waves of the dataset. Nevertheless, this study has a few limitations. First, cognitive function was not measured through clinical assessment tools such as a clinical dementia rating scale or neuropsychological battery [82,83]. Second, we could not include changes in occupation, duration of each occupation, or premorbid intelligence [84] owing to the lack of such information in the dataset.

## 5. Conclusions

This is the first study to investigate and quantify the gender-specific association of the risk of cognitive impairment across diverse occupations, including unpaid work, in the middle-age population using the dataset of a national longitudinal study. This study, as in the previous study [20], showed a difference in the adjusted risk of cognitive impairment across unpaid and paid occupations. Contrary to the previous study, the adjusted risk of cognitive impairment was similar between genders, while the adjusted association between occupation and the risk of cognitive impairment differed between genders. In terms of the predicted probability, in men, the adjusted risk of cognitive impairment in the retired category was more than twice the risk in the professionals and related workers category; however, in women, the risk in the homemakers category was more than five times that in the professionals and related workers category. From a policy perspective, we emphasize targeting high-risk groups such as retired people in men and life-long homemakers in women. Further research would be interesting to test if these results and corresponding suggestions derived from the Korean dataset are effective in other socio-cultural or economic development settings.

## Figures and Tables

**Table 1:** Sample characteristics at baseline (Wave 1).

Characteristics	Men	Women	*p* ^h^
Cognitive function ^a^: mean (SD) ^b^, median	27.9 (2.8); 29.0	27.1 (3.4); 28.0	<0.001
Age, years: mean (SD) ^b^; median	54.1 (5.8); 54.0	53.9 (5.9); 53.0	0.222
Non-married ^c^	6.6%	15.6%	<0.001
Religion, yes	45.2%	64.5%	<0.001
Reside in rural area	18.1%	18.7%	0.487
Educational level			<0.001
Elementary school or less	18.3%	38.4%	
Middle school or high school	60.1%	54.2%	
College or higher	21.6%	7.4%	
Occupation			<0.001
Homemakers	11.0%	50.9%	
Unemployed	6.0%	4.9%	
Retired	7.5%	9.6%	
Managers	10.8%	1.6%	
Professionals and related workers	5.1%	1.8%	
Clerks	8.5%	2.2%	
Service workers	3.3%	8.6%	
Sales workers	6.7%	6.3%	
Skilled agricultural, forestry and fishery workers	6.1%	3.8%	
Craft and related trades workers	9.9%	2.0%	
Plant and machine operators and assemblers	11.3%	1.5%	
Elementary workers and armed forces	14.0%	6.9%	
Household income ^d^			<0.001
Lower half	43.8%	50.4%	
Higher half	50.4%	43.7%	
Unreported	5.8%	5.9%	
House renter	21.9%	23.4%	0.352
Smoker, yes	46.4%	2.6%	<0.001
Alcohol drinker, yes	71.3%	23.8%	<0.001
Active routine physical exercise	45.0%	41.9%	0.074
Obese ^e^	24.4%	24.9%	0.938
Have chronic disease ^f^	29.1%	28.6%	0.555
Have depressive symptom ^g^	17.6%	25.8%	<0.001
Number of observations	2619	3246	

^a^ Cognitive function was based on the Korean Mini-Mental State Examination scores. ^b^ SD denotes standard deviation. ^c^ Non-married included never married, separated, widowed, or divorced. ^d^ Household income was adjusted for household size for each wave. ^e^ Obese was defined as a body mass index of at least 25 kg/m^2^. ^f^ Chronic diseases included hypertension, diabetes, stroke, angina, myocardial infarction, chronic pulmonary diseases, and any type of cancer. ^g^ Depressive symptom was defined as a score of 4 or more on the 10-item short form of the Center for Epidemiologic Studies Depression Scale. ^h^ *p*-values for both cognitive function and age were obtained from a nonparametric equality-of-medians test, and they, for all categorical variables, were from the design-based chi-squared test.

**Table 2:** Prevalence rate (%) of cognitive impairment by occupation category for each gender at baseline (Wave 1).

Occupation	Men		Women	
	Rate	(95% CI)	Rate	(95% CI)
Overall	5.7	(4.8–6.6)	11.5	(10.5–12.7)
Chi-squared test, *p*-value	<0.001			
Occupation				
Homemakers	9.2	(6.2–13.4)	14.1	(12.5–16.0)
Unemployed	8.1	(4.6–14.0)	9.0	(5.4–14.8)
Retired	14.5	(10.4–19.9)	12.8	(9.3–17.2)
Managers	2.9	(1.4–6.1)	-	-
Professionals and related workers	3.1	(1.4–6.8)	-	-
Clerks	2.3	(1.0–5.0)	2.8	(0.7–10.8)
Service workers	4.8	(1.5–13.9)	7.2	(4.7–10.7)
Sales workers	3.5	(1.4–8.2)	3.1	(1.6–6.1)
Skilled agricultural, forestry and fishery workers	7.0	(3.9–12.3)	22.3	(15.5–31.0)
Craft and related trades workers	4.6	(2.4–8.6)	4.2	(1.2–13.4)
Plant, machine operators and assemblers	3.5	(1.8–7.0)	8.0	(2.9–19.9)
Elementary workers and armed forces	7.3	(5.0–10.6)	13.8	(9.9–18.9)
Chi-squared test, *p*-value	<0.001		<0.001	
Number of observations	2619		3246	

Prevalence estimation and tests were performed by considering a complex sampling design. CI denotes the confidence interval.

**Table 3:** Longitudinal analyses of the associations between occupation categories and cognitive impairment.

Characteristics	Model with No Covariate						Model with All-Studied Covariates					
	Men			Women			Men			Women		
	OR ^a^	(95% CI) ^b^	*p*	OR ^a^	(95% CI) ^b^	*p*	OR ^a^	(95% CI) ^b^	*p*	OR ^a^	(95% CI) ^b^	*p*
Occupation (Ref: Homemakers); Wald test, *p*-value	0.005			<0.001			0.006			<0.001		
Unemployed	0.70	(0.40–1.23)	0.213	0.57	(0.35–0.94)	0.026	0.93	(0.54–1.60)	0.785	0.64	(0.39–1.05)	0.076
Retired	1.36	(0.92–2.02)	0.121	0.78	(0.61–0.98)	0.035	1.49	(0.99–2.23)	0.054	0.72	(0.57–0.91)	0.006
Managers	0.31	(0.16–0.58)	<0.001	0.30	(0.10–0.93)	0.037	0.94	(0.49–1.78)	0.842	0.56	(0.19–1.63)	0.289
Professionals and related workers	0.11	(0.05–0.26)	<0.001	0.02	(0.01–0.11)	<0.001	0.34	(0.14–0.81)	0.015	0.10	(0.02–0.43)	0.002
Clerks	0.23	(0.11–0.47)	<0.001	0.18	(0.07–0.47)	<0.0001	0.68	(0.33–1.37)	0.277	0.37	(0.16–0.88)	0.024
Service workers	0.28	(0.12–0.68)	0.005	0.45	(0.31–0.64)	<0.001	0.52	(0.22–1.24)	0.140	0.65	(0.46–0.93)	0.018
Sales workers	0.27	(0.14–0.53)	<0.001	0.32	(0.20–0.50)	<0.001	0.52	(0.28–0.98)	0.043	0.48	(0.31–0.75)	0.001
Skilled agricultural, forestry, and fishery workers	0.46	(0.24–0.88)	0.019	1.17	(0.78–1.76)	0.454	0.55	(0.30–1.03)	0.064	0.70	(0.47–1.05)	0.083
Craft and related trades workers	0.46	(0.26–0.84)	0.011	0.31	(0.14–0.73)	0.007	0.72	(0.40–1.29)	0.269	0.50	(0.23–1.10)	0.087
Plant and machine operators and assemblers	0.42	(0.25–0.71)	0.001	0.21	(0.08–0.57)	0.002	0.68	(0.40–1.16)	0.157	0.30	(0.11–0.81)	0.018
Elementary workers and armed forces	0.64	(0.42–0.98)	0.038	0.75	(0.56–1.00)	0.047	0.81	(0.53–1.23)	0.313	0.67	(0.51–0.89)	0.006
Age (Ref: Mean value)							1.05	(1.02–1.08)	0.001	1.07	(1.04–1.09)	<0.001
Non-married ^c^ (Ref: Married)							1.42	(0.88–2.31)	0.153	1.14	(0.88–1.48)	0.317
Religion, yes (Ref: No)							0.75	(0.58–0.98)	0.035	0.76	(0.64–0.90)	0.001
Reside in rural area (Ref: Reside in an urban area)							0.85	(0.58–1.25)	0.413	1.25	(0.98–1.60)	0.068
Educational level (Ref: Elementary school or less)												
Middle school or high school							0.27	(0.19–0.38)	<0.001	0.23	(0.19–0.29)	<0.001
College or higher							0.14	(0.00–0.23)	<0.001	0.09	(0.05–0.18)	<0.001
Household income ^d^, higher half (Ref: Lower half or unreported)							0.82	(0.64–1.06)	0.128	0.82	(0.68–0.98)	0.026
House renter (Ref: Owner)							0.89	(0.62–1.27)	0.512	1.30	(1.02–1.65)	0.036
Smoker (Ref: Non-smoker)							0.75	(0.57–0.98)	0.036	1.03	(0.55–1.94)	0.922
Alcohol drinker (Ref: Non-alcohol-drinker)							0.69	(0.52–0.93)	0.013	0.67	(0.53–0.85)	0.001
Active routine physical exercise (Ref: Not active)							0.65	(0.49–0.86)	0.003	0.68	(0.57–0.82)	<0.001
Obese ^e^ (Ref: Not obese)							0.88	(0.64–1.21)	0.430	1.12	(0.91–1.36)	0.283
Have chronic disease ^f^ (Ref: No)							1.30	(0.98–1.73)	0.068	1.20	(0.99–1.45)	0.069
Have depressive symptom ^g^ (Ref: No)							2.57	(2.02–3.28)	<0.001	1.88	(1.60–2.22)	<0.001
Number of observations												

The effect of a continuous variable, age, was assessed as one unit offset from the mean. All values were estimated using a complex sampling design. All characteristics were considered to be time-dependent. ^a^ OR, odds ratio. ^b^ CI, confidence interval. ^c^ Non-married included never married, separated, widowed, or divorced. ^d^ Household income was adjusted for household size for each wave. ^e^ Obese was defined as a body mass index of at least 25 kg/m^2^. ^f^ Chronic diseases included hypertension, diabetes, stroke, angina, myocardial infarction, chronic pulmonary diseases, and any type of cancer. ^g^ Depressive symptom was defined as a score of 4 or more on the 10-item short form of the Center for Epidemiologic Studies Depression Scale.

## Supplementary Materials

The following are available online at https://www.mdpi.com/1660-4601/17/21/7749/s1, Table S1: Sample characteristics at waves 2 to 7 by gender.
