# Supplementary material for: Which Occupation is Highly Associated with Cognitive Impairment? A Gender-Specific Longitudinal Study of Paid and Unpaid Occupations in South Korea
Source: Int J Environ Res Public Health. 2020 Oct 23;17(21):7749. doi: 10.3390/ijerph17217749 (PMC7660334; doi:10.3390/ijerph17217749)
Supplement: Supplementary file 1 [file ijerph-17-07749-s001.pdf]

Supplementary File

Table S1. Sample characteristics at waves 2 to 7 by gender.

**Characteristics**	**Men**						**Women**					
	**Wave 2 (2008)**	**Wave 3 (2010)**	**Wave 4 (2012)**	**Wave 5 (2014)**	**Wave 6 (2016)**	**Wave 7 (2018)**	**Wave 2 (2008)**	**Wave 3 (2010)**	**Wave 4 (2012)**	**Wave 5 (2014)**	**Wave 6 (2016)**	**Wave 7 (2018)**
Cognitive function [a]: mean (SD) [b]	27.9 (2.5)	27.9 (2.8)	28.0 (2.7)	28.0 (2.8)	28.1 (2.6)	27.8 (3.5)	27.2 (3.1)	27.2 (3.1)	27.4 (3.0)	27.4 (3.1)	27.6 (3.0)	27.5 (3.1)
Age, years: mean (SD) [b]	55.3 (5.2)	56.5 (4.7)	57.5 (4.1)	58.5 (3.5)	59.6 (2.9)	60.6 (2.3)	54.8 (5.2)	56.1 (4.6)	57.2 (4.1)	58.3 (3.5)	59.3 (2.9)	60.3 (2.3)
Non-married [c]	7.0%	6.7%	7.2%	6.7%	7.0%	8.6%	14.7%	13.8%	13.5%	13.8%	13.6%	13.8%
Religion, yes	40.3%	36.5%	36.9%	31.3%	30.4%	28.1%	59.3%	57.0%	58.6%	50.2%	49.4%	42.6%
Reside in rural area	20.0%	20.6%	20.1%	19.3%	18.5%	19.9%	19.7%	21.4%	20.7%	20.7%	19.9%	20.3%
Educational level												
Elementary school or less	17.2%	15.0%	13.3%	11.1%	10.1%	9.4%	34.3%	32.2%	30.4%	26.6%	22.1%	19.2%
Middle school or high school	61.9%	64.3%	64.6%	64.6%	65.1%	66.1%	58.6%	60.5%	62.5%	65.7%	69.3%	71.2%
College or higher	21.0%	20.7%	22.2%	24.2%	24.8%	24.5%	7.1%	7.3%	7.2%	7.8%	8.7%	9.6%
Occupation												
Homemakers	10.1%	7.6%	7.7%	11.8%	6.9%	18.4%	40.1%	38.0%	36.8%	36.6%	35.5%	46.2%
Unemployed	4.2%	3.7%	3.4%	2.0%	2.9%	3.1%	4.1%	2.4%	2.8%	1.8%	2.1%	1.6%
Retired	7.8%	8.7%	10.5%	6.8%	11.0%	13.3%	13.4%	13.0%	15.9%	18.9%	19.3%	18.9%
Managers	7.4%	6.1%	6.0%	6.6%	6.2%	3.5%	0.5%	0.6%	0.7%	0.8%	0.9%	0.5%
Professionals and related workers	7.9%	7.7%	7.2%	6.9%	6.5%	5.9%	2.7%	3.2%	3.2%	2.4%	2.3%	2.0%
Clerks	7.4%	7.2%	6.7%	6.4%	5.0%	3.8%	2.3%	2.2%	1.7%	1.7%	2.0%	1.8%
Service workers	4.1%	4.3%	3.9%	4.2%	5.1%	4.1%	10.5%	10.6%	10.4%	10.2%	10.8%	9.3%
Sales workers	6.8%	6.8%	6.3%	6.1%	6.1%	5.3%	8.0%	7.9%	7.3%	6.7%	6.4%	4.9%
Skilled agricultural, forestry and fishery workers	7.3%	7.9%	8.0%	7.1%	6.9%	5.4%	4.9%	5.7%	4.9%	4.6%	4.5%	3.4%
Craft and related trades workers	10.7%	11.4%	12.4%	13.1%	12.3%	12.2%	1.9%	2.2%	2.5%	2.1%	2.1%	2.0%
Plant and machine operators and assemblers	11.9%	12.5%	12.4%	12.4%	12.8%	10.9%	1.2%	1.1%	1.2%	1.4%	1.3%	1.2%
Elementary workers and armed forces	14.7%	16.2%	15.7%	16.6%	18.3%	14.1%	10.4%	13.1%	12.7%	12.9%	13.0%	8.3%
Household income [d]												

Lower half	46.0%	47.6%	46.1%	46.5%	45.5%	44.7%	52.6%	53.7%	52.9%	53.7%	55.2%	54.9%
Higher half	53.2%	52.1%	53.7%	53.4%	54.5%	54.9%	46.5%	46.2%	47.0%	45.8%	44.7%	44.9%
Unreported	0.9%	0.3%	0.2%	0.1%		0.3%	0.9%	0.2%	0.2%	0.5%	0.1%	0.2%
House renter	19.3%	18.6%	18.3%	18.0%	18.2%	17.9%	19.6%	18.8%	17.7%	17.1%	17.2%	15.4%
Smoker, yes	45.8%	46.5%	44.2%	38.7%	32.2%	29.9%	2.3%	2.3%	1.9%	1.6%	1.5%	1.2%
Alcohol drinker, yes	71.0%	70.1%	69.2%	68.9%	68.7%	69.2%	25.4%	25.6%	25.8%	23.7%	23.4%	24.7%
Active routine physical exercise	41.1%	37.8%	39.4%	33.7%	37.8%	34.1%	39.9%	37.9%	38.6%	34.6%	37.2%	35.4%
Obese [e]	21.8%	21.8%	24.1%	22.9%	24.8%	24.7%	24.0%	26.0%	25.0%	25.1%	25.4%	24.8%
Have chronic disease [f]	33.5%	35.3%	39.0%	42.0%	42.4%	43.9%	31.8%	36.6%	39.4%	41.3%	42.1%	42.6%
Have depressive symptom [g]	31.7%	31.8%	29.9%	28.5%	24.7%	28.5%	40.4%	37.5%	32.9%	31.8%	31.6%	32.6%
Number of observations	1,945	1,550	1,253	1,023	802	608	2,409	1,957	1,631	1,367	1,122	871

[a] Cognitive function was based on the Korean Mini-Mental State Examination scores. [b] SD denotes standard deviation. [c] Non-married included never married, separated, widowed, or divorced. [d] Household income was adjusted for household size for each wave. [e] Obese was defined as the body mass index of at least 25. [f] Chronic diseases included hypertension, diabetes, stroke, angina, myocardial infarction, chronic pulmonary diseases, and any type of cancer. [g] Depressive symptom was defined as a score of 4 or more on the 10-item short form of the Center for Epidemiologic Studies Depression Scale.
